# Association of plasminogen activator inhibitor-1 and tissue plasminogen activator with type 2 diabetes and metabolic syndrome in Malaysian subjects

**DOI:** 10.1186/1475-2840-10-23

**Published:** 2011-03-18

**Authors:** Zaid Al-Hamodi, Ikram S Ismail, Riyadh Saif-Ali, Khaled A Ahmed, Sekaran Muniandy

**Affiliations:** 1 Department of Molecular Medicine, Faculty of Medicine, University of Malaya, 50603 Kuala Lumpur, Malaysia; 2 Department of Medicine, University of Malaya Medical Centre, University of Malaya, 50603 Kuala Lumpur, Malaysia; 3 Department of Biochemistry, Faculty of Medicine, Sana'a University, Yemen; 4 Faculty of Dentistry, Ibb University, Yemen

## Abstract

**Background:**

Increased plasma plasminogen activator inhibitor-1 (PAI-1) activity and decreased tissue plasminogen activator (tPA) activity could be considered a true component of the metabolic syndrome (MetS) associated with an increased risk of developing cardiovascular diseases (CVD) and fibrinolytic abnormalities. The aim of this study was to investigate the association of tPA and its inhibitor PAI-1 with type 2 diabetes (T2D) and MetS and interrelationship between PAI-1and tPA activities and antigens in Malaysian T2D and normal subjects.

**Methods:**

The plasma activities and antigens of PAI-1 and tPA and the levels of the tPA/PAI-1 complex as well as serum insulin, parameter of the coronary risk panel and plasma glucose at fasting state were studied in 303 T2D subjects (227 with MetS and 76 without MetS), 131 normal non-diabetic non-metabolic subjects and 101 non-diabetic MetS subjects.

**Results:**

The PAI-1 activity was higher in subjects with T2D with MetS (P = 9.8 × 10^-19^) and non-diabetic subjects with MetS (P = 3.0 × 10^-15^), whereas the tPA activity was lower in T2D with MetS (P = 0.003) as compare to normal subjects. Plasma tPA antigen levels were higher in subjects with T2D with MetS (P = 8.9 × 10^-24^), T2D without MetS (P = 1.3 × 10^-13^) and non-diabetic MetS subjects (P = 0.002). The activity and antigen of PAI-1 in normal subjects were related to insulin resistance (P = 2.2 × 10^-4^; 0.007). Additionally, the PAI-1 activity was associated with an increased waist circumference (P = 2.2 × 10^-4^) and decreased HDL-c (P = 0.005), whereas the tPA activity was associated with decreased FBG (P = 0.028). The highest correlation was between PAI-1 activity and its antigen (R^2 ^= 0.695, P = 1.1 × 10^-36^) in diabetic subjects. The tPA activity negatively correlated with its antigen (R^2 ^= -0.444, P = 7.7 × 10^-13^) in normal subjects and with the PAI-1 activity and antigen (R^2 ^= -0.319, P = 9.9 × 10^-12^; R2 = -0.228, P = 3.4 × 10^-6^) in diabetic subjects.

**Conclusions:**

PAI-1 and tPA activities and antigens were associated with diabetes and MetS parameters in Malaysian subjects.

## Background

Central obesity, hyperglycaemia, high triglycerides (TG), low high-density lipoprotein cholesterol (HDL-c) and hypertension, all are well-documented risk factors for type 2 diabetes (T2D) and cardiovascular diseases(CVD) [1]. The constellation of these metabolic abnormalities (known as a metabolic syndrome (MetS) or insulin resistance syndrome) increases the risk of T2D and CVD. The number of individuals with the MetS has increased globally during the past two decades, and this increase is associated with the worldwide epidemic of obesity and diabetes [2]. Obesity and physical inactivity are the driving forces for MetS and a person with MetS has 5-fold higher relative risk to develop diabetes [3] and 2.5 fold higher to develop CVD [4,5]. Overweightedness and obesity lead to adverse effects on blood pressure, cholesterol, TG and impaired glucose tolerance (IGT) [6].

Plasminogen activator inhibitor-1 (PAI-1) is the primary physiological inhibitor of endogenous fibrinolysis that acts via inhibition of the tissue plasminogen activator (tPA) and the urokinase type activator (uPA), often leading to fibrin accumulation in basement membranes and interstitial tissues [7-9]. Elevations in plasma PAI-1 appear to compromise normal fibrin clearance mechanisms and promote thrombosis. The plasminogen activators (t-PA and u-PA) convert plasminogen to plasmin, which is involved in fibrinolysis, tissue remodelling and cell migration [10]. In addition to its role in intravascular fibrinolysis, PAI-1 is also involved in cell-associated proteolysis, cell migration, and tissue remodelling playing a role in pathological processes such as cancer cell invasion, metastasis and inflammation [11,12]. The majority of tPA in the blood is bound to its primary inhibitor, PAI-1[13].

In large epidemiological studies elevated plasma PAI-1 has been demonstrated in various subgroups as an important feature of T2D and MetS [14-20] and this elevation may contribute to a thrombotic tendency [7-9,15,21]. This elevation precedes coronary artery disease [22] and even predicts the occurrence of first acute myocardial infarction and reinfarction [23-25]. Remarkably, the predictive ability of PAI-1 disappears after adjustment for markers of the MetS [26,27], suggesting that the MetS is a prerequisite to high plasma PAI-1 levels in patients prone to atherothrombosis. Moreover, it has been hypothesized that PAI-1 participates in the development of key features of the MetS. The circulating PAI-1 levels are positively associated with obesity and insulin resistance [28-30].

tPA activity may be an independent and early marker for asymptomatic lower extremity arterial disease in T2D [18,31]. Plasma tPA activities and the capacity of endothelial cells to secret tPA in response to a fibrinolytic stimulus were also reported to be decreased in adults with diabetes [15]. Elevated plasma tPA antigens have been reported to be associated with insulin resistance, T2D, and obesity [14,18] and increased risk of CHD [32].

The aim of this study was to investigate the association of the plasma activities and antigens of PAI-1 and tPA, with T2D and MetS and to study the relationship between activities and antigens of PAI-1 and tPA.

## Materials and methods

### Subjects and data collection

This study involved diabetic patients with and without MetS and non-diabetic subjects with MetS receiving treatment at the University Malaya Medical Centre (UMMC), Kuala Lumpur. Normal subjects without diabetes and MetS (the control group) in Klang Valley, Kuala Lumpur were recruited. The study was approved by the Medical Ethics Committee of University Malaya Medical Centre. Written informed consent was obtained from each subject. Patients with acute or chronic infections, severe medical conditions (malignancy, renal failure, liver cirrhosis, connective tissue disease, and chronic congestive heart failure) and pregnant women were excluded from the study.

Blood pressure (BP) measurements were taken from each patient's right arm in the seated position by using an Omron IntelliSense Automatic Blood Pressure Monitor after 10 min of rest in a quiet room in the morning. Two to three successive BP readings were obtained at 5-min intervals and averaged. Body weight and height were measured in the fasting state without shoes in the morning, and BMI was computed as weight in kilograms (kg) divided by height in meters squared (m^2^). Waist circumference was measured midway between the lower rib margin and the superior iliac spine at the end of gentle expiration in a standing position.

Fasting venous blood (10ml) was collected from each subject within a 2-hour window (8:00 to 10:00 AM) after 15 minutes rest, because of the diurnal variation of plasma PAI-1 [33]. The collected blood immediately taken into four labelled Vacutainer tubes, 0.109M trisodium citrate (for tPA and PAI-1 antigens and tPA/PAI-1 complex analysis), acidified 0.5M sodium citrate (for tPA and PAI-1 activities), sodium fluoride (for glucose measurement) and plain tubes (for insulin and lipid profile). The plasma/serum of trisodium citrate and plain tubes were separated gently within 30 minutes by using Allerga R X-12R centrifuge (Beckman Coulter. Inc US) for a 15 min at 2500-3000 × g at 4°C. Then the plasma/serum was separated into corresponding micro tubes and immediately kept at -80°C until analysis.

### Biochemical Analyses

Serum TG, HDL-c and plasma glucose (FPG) were measured by an automated analyzer Dimension^® ^RxL Max^® ^Integrated Chemistry System. Insulin was measured by ADVIA Centaur assay XP Immunoassay System (Siemens Healthcare Diagnostics Inc. Deerfield, IL USA). All these investigations were done at Clinical Diagnostic Laboratory of the University Malaya Medical Centre (UMMC), Kuala Lumpur. Insulin resistance (IR) and Insulin sensitivity (IS) were calculated using the Homeostasis Model Assessment (HOMA2) Calculator v2.2 which is available from Oxford Centre for Diabetes, Endocrinology and Metabolism.

Plasma PAI-1 antigen was measured by TintElize^® ^PAI-1 antigen ELISA test from Biopool (Trinity Biotech Inc. USA, Jamestown, NY). PAI-1 activity, tPA activity and antigen and tPA/PAI-1complex were measured by PAI-1 activity, tPA activity, tPA total antigen, tPA/PAI-1 complex human assays respectively, (Molecular Innovations, Inc. Peary Court, Novi, MI USA) according to the manufacturer's instructions. Plates were read at 450nm except the PAI-1 antigen plate which was at 490nm using a microplate reader (BioRad, USA).

### Statistical Analyses

The results were analyzed by the SPSS 11.5 (Social Package of Statistical Science) computer program by LEAD Technologies; Inc. USA). The missing data were listwise deleted (when any of the variables were missing, the entire observation was omitted from the analysis). The age, body mass index (BMI), waist, systolic blood pressure (SBP), diastolic blood pressure (DBP), TG, HDL-c, FBS, insulin, IS, IR, PAI-1 and tPA activities and antigens and tPA/PAI-1 complex were log transformed because they were not normally distributed. These parameters means and 95% confidence intervals were transformed back and reported as geometric means. The association of PAI-1 and tPA activities and antigens and tPA/PAI-1 complex with T2D and MetS were assessed by univariate analyses (general linear model) adjusted for age, gender and race as covariates. The association of fibrinolytic PAI-1 and tPA activities and antigens with MetS parameters; (BMI, waist, SBP, DBP, TG, HDL-c, FBS, insulin, IS and HOMA (IR) (dependent variables) were analysed by hierarchical linear regression adjusted for age, gender and race as covariates in normal subjects. The interrelationship between fibrinolytic parameters; PAI-1 and tPA activities and antigens and tPA/PAI-1 complex in normal and T2D subjects were first evaluated by linear regression. This evaluation showed that (BMI, HDL-c and IR), (HDL-c and IR), (FBS), (Age and IS) and (BMI) were significantly associated with PAI-1 activity, PAI-1 antigen, tPA activity, tPA antigen and tPA/PAI-1 complex respectively. Then the correlation of fibrinolytic parameters with each other re-evaluated by hierarchical linear regression controlled for those corresponding confounder in addition to age, gender and race.

## Results

Of five hundred normal subjects freely respondents, only 190 were recruited for the study. After biochemical tests and application of IDF criteria for MetS diagnosis [34], 131 subjects were revealed as normal without diabetes and MetS (the control group). One hundred and one subjects out of 142 participants under treatment for hyperlipidaemia and/or hypertension at University Malaya Medical Centre (UMMC) were diagnosed as non-diabetic MetS subjects. Three hundred and three subjects who were previously diagnosed as T2D were participated in this study. Of these, 227 had MetS and 76 did not have MetS as defined by the IDF criteria for MetS diagnosis.

The demography and biochemical parameters of the subjects are shown in table 1. The fibrinolytic parameters, PAI-1 and tPA activities and antigens and tPA/PAI-1 complex assessed by general linear model (univariate) are shown in table 2. In general, the plasma levels of PAI-1 activity and tPA antigens were higher in T2D patients compared to normal subjects (P = 1.7 × 10^-8^, 3.0 × 10^-24 ^respectively) whereas the PAI-1 antigen and tPA activity (P = 0.01, 0.03 respectively) were lower in T2D. There was no difference in tPA/PAI-1 complex between T2D and control subjects. On the other hand, the plasma PAI-1 activity was higher in non-diabetic MetS (P = 3.0 × 10^-15^, 1.5 × 10^-18^) and T2D with MetS subjects (P = 9.8 × 10^-19^, 1.8 × 10^-21^) compared to normal and T2D without MetS subjects respectively. Nevertheless, there was no difference in PAI-1 activity between the normal and T2D without MetS subjects and between non-diabetic MetS and T2D with MetS subjects (Table 2). However, the PAI-1 antigen was not significantly higher in non-diabetic MetS and significantly lower in T2D without MetS (P = 2.2 × 10^-9^) compared to normal subjects. The results further showed that PAI-1 antigen was lower in both T2D without MetS (P = 6.9 × 10^-13^) and T2D with MetS (P = 0.02) compared to non-diabetic MetS subjects. Besides, it was higher in T2D with MetS (P = 5.1 × 10^-10^) compared to T2D without MetS subjects. The tPA activity was lower in T2D with MetS compared to normal, non-diabetic MetS and T2D without MetS groups (P = 0.003, 0.002, 6.7 × 10^-5^) respectively. On the other hand, there were no differences in the tPA activities between the other groups. In contrast, the tPA antigen was higher in non-diabetic MetS, T2D without MetS and T2D with MetS compared to control group (P = 0.002, 1.3 × 10^-13^, 8.9 × 10^-24^) respectively. In addition, the tPA antigen was higher in T2D without MetS and T2D with MetS compared to non-diabetic MetS group (P = 1.3 × 10^-6^, 1.3 × 10^-12^) respectively. Further the results showed that tPA/PAI-1 complex was lower in the T2D without MetS compared to normal (P = 0.04), non-diabetic MetS (P = 0.02) and T2D with MetS (p = 0.001) groups while there were no differences in the tPA/PAI-1 complex between the other groups.

**Table 1:** Demography and Biochemical parameters among normal, non-diabetic metabolic syndrome and type 2 diabetes with and without metabolic syndrome groups

Parameters		Normal (n = 131)	Non-diabetic MetS (n = 101)	Type 2 diabetes	
				
				without MetS (n = 76)	with MetS (n = 227)
**Gender%**	**Male/Female**	18.5/29.2	23.9/14.8	17.7/11.3	39.9/44.7
	**Malay**	25.9	17.5	13.7	43.0
**Races %**	**Chinese**	36.1	22.1	14.8	27.0
	**Indian**	12.1	18.8	14.8	54.4
**Age (yrs)**		47.2(44.9-49.7)	52.1(50.0-54.3)	49.8(47.6-52.1)	51.4(50.5-52.4)
	**p-value**		^a^***0.003***	^a^0.349	^a^***0.002***
**Body Mass Index (kg/m2)**		22.7(22.1-23.3)	27.2(26.4-28.0)	23.34(22.5-24.3)	29.8(29.2-30.3)
	**p-value**		^a^***3.8 × 10^-13^***	^a^0.515, ^b^***6.2 × 10^-10^***	^a^***3.8 × 10^-13^, ^b^3.2 × 10^-6^, ^c^3.8 × 10^-13^***
**Waist circumference (cm)**		79.2(77.4-81.1)	94.5(92.8-96.3)	82.9(80.7-85.2)	98.6(97.4-99.7)
	**p-value**		^a^***3.8 × 10^-13^***	^a^***0.02*, **^b^***4.2 × 10^-13^***	^a^***3.8 × 10^-13^, ^b^0.006, ^c^3.8 × 10^-13^***
**Diastolic Blood Pressure (mmHg)**		80.0(78.4-81.5)	85.6(83.6-87.6)	79.3(77.1-81.5)	83.1(81.7-84.5)
	**p-value**		^a^***1.7 × 10^-4^***	^a^0.966, ^b^***2.3 × 10^-4^***	^a^***0.02***, ^b^***0.004***, ^c^***0.02***
**Systolic Blood Pressure (mmHg)**		130(127-134)	139(136-143)	129(125-134)	136(134-139)
	**p-value**		^a^***0.002***	^a^*0.965*, ^b^***0.002***	^a^***0.02***, ^c^***0.02***
**Triglyceride (mmol/l)**		1.08(1.00-1.17)	1.59(1.46-1.72)	1.38(1.19-1.57)	1.78(1.66-1.91)
	**p-value**		^a^***1.6 × 10^-6^***	^a^***0.02***	^a^***3.6 × 10^-13^, ^c^0.001***
**High Density Lipoprotein (mmol/l)**		1.54(1.48-1.61)	1.26(1.20-1.31)	1.22(1.15-1.29)	1.15(1.11-1.18)
	**p-value**		^a^***1.2 × 10^-10^***	^a^***2.6 × 10^-12^***	^a^***4.8 × 10^-13^, ^b^0.008***
**Fasting Blood Glucose (mmol/l)**		5.02(4.93-5.10)	5.36(5.23-5.49)	8.05(7.41-8.74)	7.74(7.39-8.11)
	**p-value**		0.24^a^	^a^***3.8 × 10^-13^, ^b^3.8 × 10^-13^***	^a^***3.8 × 10^-13^, ^b^3.8 × 10^-13^***
**Insulin (pmol/l)**		45.7(41.7-50.1)	77.3(69.6-85.8)	63.7(52.9-76.5)	120(110-131)
	**p-value**		^a^***7.3 × 10^-9^***	^a^***0.002***	^a^***4.1 × 10^-13^, ^b^5.0 × 10^-8^, ^c^1.4 × 10^-12^***
**Insulin resistance (IR)**		0.91(0.83-1.01)	1.53(1.37-1.69)	1.49(1.24-1.77)	2.59(2.38-2.81)
	**p-value**		^a^***1.0 × 10^-6^***	^a^***3.2 × 10^-5^***	^a^***4.1 × 10^-13^, ^b^2.5 × 10^-12^, ^c^3.8 × 10^11^***

The result presented as geometric mean and 95% confidence interval of mean, ^a^vs control group: ^b^vs non-diabetic metabolic syndrome group: ^c^vs type 2 diabetes without metabolic syndrome which evaluated by ANOVA. Bolt values are significant. MetS: metabolic syndrome.

**Table 2:** Comparison of fibrinolytic PAI-1 and tPA between normal, non-diabetic metabolic syndrome, type 2 diabetes with and without metabolic syndrome and total type 2 diabetes groups

Parameters	Normal (n = 131)	Non-diabetic MetS (n = 101)	Type 2 diabetes		Total Type 2 diabetes (n = 303)
				
			without MetS (n = 76)	with MetS (n = 227)	
**PAI-1 activity (IU/ml)**	12.8(11.1-14.7)	28.7(25.1-32.8)	11.0(9.37-12.9)	29.0(26.1-32.1)	21.7(19.7-23.9)
**p-value**		^a^***3.0 × 10^-15^, ^c^1.5 × 10^-18^***	^a^0.167	^a^***9.8 × 10^-19^, ^c^1.8 × 10^-21^***	^a^***1.7 × 10^-8^, ^b^0.02***
**PAI-1 antigen (ng/ml)**	30.2(27.1-33.7)	33.0(29.7-36.6)	18.2(16.2-20.6)	28.4(26.5-30.5)	25.4(23.9-27.1)
**p-value**		^a^0.259	^a^***2.2 × 10^-9^, ^b^6.9 × 10^-13^***	^b^***0.02***, ^c^***5.1 × 10^-10^***	^a^***0.01***, ^b^***4.7 × 10^-5^***
**tPA activity (U/ml)**	3.49(3.11-3.92)	3.51(3.14-3.91)	3.91(3.42-4.47)	2.82(2.58-3.07)	3.11(2.89-3.34)
**p-value**		^a^0.965	^a^0. 206	^a^***0.003***, ^b^***0.002***, ^c^***6.7 × 10^-5^***	^a^***0.03***, ^b^***0.03***
**tPA antigen (ng/ml)**	4.62(4.10-5.21)	5.99(5.35-6.70)	9.15(8.04-10.4)	9.85(9.14-10.6)	9.67(9.07-10.31)
**p-value**		^a^***0.002***	^a^***1.3 × 10^-13^, ^b^1.3 × 10^-6^***	^a^***8.9 × 10^-24^, ^b^1.3 × 10^-12^***	^a^***3.0 × 10^-24^, ^b^1.7 × 10^-12^***
**tPA/PAI-1 complex (ng/ml)**	3.50(2.89-4.23)	3.57(2.97-4.30)	2.59(2.09-3.20)	3.91(3.46-4.42)	3.52(3.17-3.92)
**p-value**		^a^0.879	^a^***0.04***, ^b^***0.02***	^a^0.336, ^c^***0.001***	^a^0.94

The result presented as geometric mean and 95% confidence interval of mean adjusted for age, gender and race, ^a^vs control group: ^b^vs non-diabetic metabolic syndrome group: ^c^vs type 2 diabetes without metabolic syndrome which evaluated by univariate (General Linear Model). Bolt values are significant. MetS: metabolic syndrome.

The association of fibrinolytic variables (PAI-1 and tPA activities and antigens) with MetS parameters was evaluated in normal subjects, and the results are depicted in table 3. The activity and antigen of PAI-1 associated with increased insulin levels (b = 1.941, P = 1.2 × 10^-4^; b = 1.614, P = 0.015), insulin resistance (b = 1.393, P = 2.2 × 10^-4^; b = 1.318, P = 0.007), and decreased insulin sensitivity (b = -1.963, P = 1.1 × 10^-4^; b = -1.637, P = 0.012). Furthermore, the PAI-1 activity associated with an increased waist circumference (b = 1.159, P = 2.2 × 10^-4^) and BMI (b = 1.199, P = 0.001)) and decreased HDL-c levels (b = -1.125, P = 0.005.). Although the tPA activity counteracts the PAI-1 activity which associated with decreased FBG (b = -1.079, P = 0.028), the tPA antigen showed no association with MetS parameters.

**Table 3:** Association of fibrinolytic PAI-1 and tPA with metabolic syndrome parameters among normal subjects (n = 131)

Metabolic syndrome Parameters	PAI-1 activity b (P-value)	PAI-1 antigen b (P-value)	tPA activity b (P-value)	tPA antigen b (P-value)
**Body Mass Index (kg/m2)**	1.199 ***(0.001)***	1.079 (0.199)	-1.109 (0.104)	-1.021 (0.783)
**Waist circumference (cm)**	1.159 ***(2.2 × 10^-4^)***	1.057 (0.213)	-1.033 (0.496)	-1.035 (0.543)
**Diastolic Blood Pressure (mmHg)**	-1.038 (0.342)	-1.014 (0.763)	1.064 (0.185)	1.028 (0.607)
**Systolic Blood Pressure (mmHg)**	-1.009 (0.846)	1.007 (0.889)	1.074 (0.198)	1.084 (0.195)
**Triglyceride (mmol/l)**	1.119 (0.165)	-1.012 (0.899)	1.064 (0.558)	1.127 (0.331)
**HDL- cholesterol (mmol/l)**	-1.125 ***(0.005)***	-1.067 (0.166)	-1.076 (0.174)	-1.016 (0.807)
**Fasting Blood Sugar (mmol/l)**	1.047 (0.092)	1.079 ***(0.02)***	-1.079 ***(0.028)***	1.023 (0.583)
**Insulin (pmol/l)**	1.941 ***(1.2 × 10^-4^)***	1.614 ***(0.015)***	-1.236- (0.312)	1.288 (0.298)
**Insulin Sensitivity (%)**	-1.963 ***(1.1 × 10^-4^)***	-1.637 ***(0.012)***	1.265 (0.266)	-1.294 (0.289)
**Insulin Resistance**	1.393 ***(2.2 × 10^-4^)***	1.318 ***(0.007)***	-1.119 (0.305)	1.135 (0.313)

Result presented as b (P-value) assessed by linear regression, adjusted for age, gender and race. Bolt values are significant. b: coefficient for the relationship between the dependent variable "metabolic syndrome parameters" and the independent variable "fibrinolytic PAI-1 and tPA parameters". The positive sign of the coefficient implies a direct relationship, and the negative sing implies an inverse relationship.

The interrelationship between activities and antigens of PAI-1 and tPA and tPA/PAI-1 complex parameters were further assessed in normal and T2D groups as shown in Table 4 and 5 respectively. Among normal subjects, the tPA activity negatively correlated with its antigen (r^2 ^= -0.44, P = 7.7 × 10^-13^) and weakly correlated with tPA/PAI-1 complex (r^2 ^= -0.090, P = 0.001). In contrast, the tPA antigen positively correlated with tPA/PAI-1 complex (r^2 ^= 0.202, P = 3.5 × 10^-7^). Unlike normal subjects, there was a good correlation between fibrinolytic parameters in T2D, the PAI-1 activity well and positively correlated with its antigen (r^2 ^= 0.70, P = 1.1 × 10^-36^), and negatively correlated with tPA activity (r^2 ^= -0.32, P = 9.9 × 10^-12^). A similar correlation was shown between PAI-1 antigen and tPA activity (r^2 ^= -0.23, P = 3.4 × 10^-6^). The pattern of tPA antigen correlation with tPA/PAI-1 complex among T2D was similar to that in normal subjects, but it was a weak correlation (r^2 ^= -0.20, P = 3.5 × 10^-7^; r^2 ^= 0.06, P = 7.9 × 10^-5^).

**Table 4:** Correlation between PAI-1 and tPA activities and antigens and tPA/PAI-1 complex parameters among normal group (n = 131)

Parameters	Adjusted for	PAI-1 activity (IU/ml)	PAI-1 antigen (ng/ml)	tPA activity (U/ml)	tPA antigen (ng/ml)	tPA/PAI-1complex (ng/ml)
**PAI-1 activity**	Age, gender, race, BMI, HDL-c & HOMA (IR)	**1**	0.085(0.973)	0.051(0.276)	-0.100(0.598)	0.035(0.552)
**PAI-1 antigen**	Age, gender, race, HDL-c & HOMA (IR)		1	0.044(0.60)	0.111(0.642)	0.029***(0.03)***
**tPA activity**	Age, gender, race & FBS			1	-0.444***(7.7 × 10^-13^)***	-0.090***(0.001)***
**tPA antigen**	Age, gender, race & IS				1	0.202***(3.5 × 10^-7^)***

Result presented as adjusted r^2 ^(P-value) assessed by Linear regression. Bolt values are significant.

**Table 5:** Correlation between PAI-1 and tPA activities and antigens and tPA/PAI-1 complex parameters among type 2 diabetes group (n = 303)

Parameters	Adjusted for	PAI-1 activity (IU/ml)	PAI-1 antigen (ng/ml)	tPA activity (U/ml)	tPA antigen (ng/ml)	tPA/PAI-1 complex (ng/ml)
**PAI-1 activity**	Age, gender, race, BMI, TG & IS	1	0.70***(1.1 × 10^-36^)***	-0.32***(9.9 × 10^-12^)***	0.038(0.132)	-0.078(0.773)
**PAI-1 antigen**	Age, gender, race, waist, TG & IS		1	-0.23***(3.4 × 10^-6^)***	0.024(0.08)	0.066(0.054)
**tPA activity**	Age, gender, race, BMI, TG & IS			1	-0.100***(5.1 × 10^-5^)***	-0.071(0.054)
**tPA antigen**	Age, gender, race & HDL-c				1	0.064***(7.9 × 10^-5^)***

Result presented as adjusted r^2 ^(P-value) assessed by Linear regression. Bolt values are significant.

## Discussion

The Association of fibrinolytic parameters with T2D and metabolic syndrome were studied; a higher PAI-1 activity is associated with T2D with MetS and MetS (nondiabetic), which is characterized by the presence of abdominal obesity and insulin resistance; this result in agreement with other studies [35-40]. The plasma PAI-1 circulates in two states, active and latent. Active PAI-1 is bound to vitronectin (VN) [41,42] (the net molecular weight of PAI-1/vitronectin, ~125,000) while latent PAI-1 is unbound (MW ~50,000). The plasma vitronectin levels are increased in diabetes with nephropathy [43]. T2D subjects with renal failure were excluded from this study. However, nephropathy in T2D starts from the early stage of development diabetes. Thus the latent PAI-1 may be excreted in urine in quantities more than PAI-1/vitronectin complex, which may result in an increase in the ratio of active to latent PAI-1 in T2D compared to normal subjects. This may explain why PAI-1 antigen is lower in T2D while PAI-1 activity is higher.

The Association of fibrinolytic parameters with MetS parameters was assessed in normal (control subjects), since the diabetic and non-diabetic MetS under treatment. There was a clear trend shows increasing of PAI-1 activity with increased insulin resistance, waist circumference, BMI, and decreased HDL-c. This would also explain the non-association of PAI-1 activity in subjects with T2D but without MetS.

PA1-1 is partly synthesised in fat cells, and its activity is related to abdominal obesity as mirrored by the high waist circumference [44]. In this regard, lipid infusion in normal subjects to the levels observed in T2D, and obesity resulted in increased PAI-1 concentration by 2 fold [45]. Dysfunctions in the fibrinolytic and endothelial system precede the development of overt T2D, which increases the risk of atherothrombotic disease long before overt diabetes is evident [45-47]. In addition, PAI-1 may predict T2D independent of insulin resistance and other known risk factors for diabetes, which may be due to increase in PAI-1 activity influenced by PAI-1 gene polymorphisms, adrenal steroids and angiotensin II activity [16,17]. The PAI-1 antigen levels were low in T2D with MetS and much lower in T2D without MetS. This finding can be partially explained by the high correlation between PAI-1 activity and its antigen in T2D (but not in normal subjects). On the other hand, PAI-1 activity was remarkably high in T2D with MetS but was normal in T2D without MetS. The high concentration of tPA antigen was mainly associated with T2D without effect of MetS parameters which in agreement with Eliasson et al, study [48]. The mechanism that explains this elevation of tPA antigen in T2D is yet unclear. The PAI-1 activity is negatively correlated with tPA activity, which may reflect the low tPA activity in T2D with MetS but not in T2D without MetS. High PAI-1 antigen inhibits tPA released from the vessel walls and decreased levels of free PA, *i.e. *low tPA activity [48].

The results of this study showed the increase in the concentration of tPA antigen was not paralleled by an increase in free tPA. Therefore, plasma tPA activity was not increased and even declined modestly in T2D which in accordance with pervious study [49]. The low levels of plasma PAI-1 in T2D without MetS subjects may have reduced the combination of PAI-1 with free tPA. This leads to decrease the tPA/PAI-1 complex concentration in this group with no decline or simply an increase in free tPA levels.

MetS is estimated to be present in 75-80% of T2D patients and has been shown to influence CVD [50,51] and fibrinolytic function [52,53]. In patients with MetS but without diabetes, increased measures of atherosclerosis [54-56] and a higher incidence of cardiovascular events has been observed [52,57]. This has partly been attributed to impaired fibrinolytic function [52] due to altered regulation of PAI-1 and tPA [53,58,59]. However, treatment by a PAI-1 inhibitor improved histological remodelling of myocardium and arteries with suppression of inflammation and thrombus formation [60]. Metabolic- defining criteria were significant predictors for PAI-1 activity, while none of the diabetes related factors seemed to contribute to PAI-1 activity and tPA levels. Thus, the main inhibitor of fibrinolysis was found to be severely influenced by the state of MetS.

In some studies, decreased fibrinolysis, as measured by high PAI-1 activity, has been associated with cardiovascular events [61,62]. High levels of tPA antigen independently predict cardiovascular events both in a healthy population [62,63] and in patients with prevalent coronary disease [6,64]. Therefore, these results also support the hypothesis that several abnormal metabolic parameters are essential for the elevation of plasma PAI-1 and reduction of tPA activities since PAI-1 is a multifunctional protein. However, more studies are needed to further assess the association between PAI-1 expression and CVD.

## Conclusions

The PAI-1 activity was pronounced in MetS and T2D with MetS whereas the tPA antigen was an indicator for T2D without effect of MetS parameters. This impaired fibrinolysis leads to a suggestion that coronary heart disease seems to start tick in non-diabetic MetS before the onset of clinical diabetes. This was confirmed in quantitative trait analysis in which PAI-1 activity was associated with MetS parameters (waist, BMI, HDL-c and insulin resistance) which are well documented as risk factors for T2D and CVD.

## Abbreviations

BMI: body mass index; CHD: Coronary heart disease; CI: confidence interval; CVD: cardiovascular disease; DBP: diastolic blood pressure; FBS: fasting blood sugar; HDL-c: high density lipoprotein cholesterol; HOMA (IR): Homeostasis Model Assessment (Insulin resistance); IDF: International Diabetes Federation; IS: Insulin sensitivity; MetS: metabolic syndrome; PAI-1: plasminogen activator inhibitor-1; SBP: systolic blood pressure; TG: triglycerides; tPA: tissue plasminogen activator; T2D: type 2 diabetes; UMMC: University Malaya medical centre; uPA: urokinase plasminogen activator.

## Competing interests

The authors declare that they have no competing interests.

## Authors' contributions

ZH collected data, performed practical and statistical analysis and drafted the manuscript; SA, performed statistical analysis, edited the manuscript and discussion. All authors read and approved the final manuscript.

## References

[B1] Eckel RH Grundy SM Zimmet PZ The metabolic syndrome Lancet 2005 365 1415 1428 10.1016/S0140-6736(05)66378-7 15836891

[B2] Zimmet P Alberti KG Shaw J Global and societal implications of the diabetes epidemic Nature 2001 414 782 787 10.1038/414782a 11742409

[B3] Grundy SM Metabolic syndrome pandemic Arterioscler Thromb Vasc Biol 2008 28 629 636 10.1161/ATVBAHA.107.151092 18174459

[B4] Arnlov J Ingelsson E Sundstrom J Lind L Impact of body mass index and the metabolic syndrome on the risk of cardiovascular disease and death in middle-aged men Circulation 2010 121 230 236 10.1161/CIRCULATIONAHA.109.887521 20038741

[B5] Prabhakaran D Anand SS The metabolic syndrome: an emerging risk state for cardiovascular disease Vasc Med 2004 9 55 68 10.1191/1358863x04vm515ra 15230489

[B6] Rassool GH Expert report on diet, nutrition and prevention of chronic diseases J Adv Nurs 2003 43 544 545 10.1046/j.1365-2648.2003.02792.x 14515830

[B7] Dellas C Loskutoff DJ Historical analysis of PAI-1 from its discovery to its potential role in cell motility and disease Thromb Haemost 2005 93 631 640 15841306 10.1160/TH05-01-0033

[B8] Kohler HP Grant PJ Plasminogen-activator inhibitor type 1 and coronary artery disease N Engl J Med 2000 342 1792 1801 10.1056/NEJM200006153422419 10853003

[B9] Lijnen HR Pleiotropic functions of plasminogen activator inhibitor-1 J Thromb Haemost 2005 3 35 45 10.1111/j.1538-7836.2004.00827.x 15634264

[B10] Kruithof EK Baker MS Bunn CL Biological and clinical aspects of plasminogen activator inhibitor type 2 Blood 1995 86 4007 4024 7492756

[B11] Dano K Behrendt N Hoyer-Hansen G Johnsen M Lund LR Ploug M Romer J Plasminogen activation and cancer Thromb Haemost 2005 93 676 681 15841311 10.1160/TH05-01-0054

[B12] Myohanen H Vaheri A Regulation and interactions in the activation of cell-associated plasminogen Cell Mol Life Sci 2004 61 2840 2858 10.1007/s00018-004-4230-9 15558213 PMC11924493

[B13] Bachmann F Colman RW HJ, Marder VJ "Plasminogen-Plasmin Enzyme Systems," Hemostasis and Thrombosis, Basic Principles and Clinical Practice LippLippincott Williams and Wilkinsincott Williams and Wilkins 2001 Philadelphia, PA 275 320

[B14] Cavallero E Dachet C Assadolahi F Martin C Navarro N Ansquer JC Corda C Foucher C Juhan-Vague I Jacotot B Micronized fenofibrate normalizes the enhanced lipidemic response to a fat load in patients with type 2 diabetes and optimal glucose control Atherosclerosis 2003 166 151 161 10.1016/S0021-9150(02)00321-0 12482562

[B15] Colwell J Altered platelet function and fibrinolysis in diabetes mellitus Endotrends 2000 7 5 8

[B16] Festa A D'Agostino R Jr Tracy RP Haffner SM Elevated levels of acute-phase proteins and plasminogen activator inhibitor-1 predict the development of type 2 diabetes: the insulin resistance atherosclerosis study Diabetes 2002 51 1131 1137 10.2337/diabetes.51.4.1131 11916936

[B17] Festa A Williams K Tracy RP Wagenknecht LE Haffner SM Progression of plasminogen activator inhibitor-1 and fibrinogen levels in relation to incident type 2 diabetes Circulation 2006 113 1753 1759 10.1161/CIRCULATIONAHA.106.616177 16585388

[B18] Glowinska B Urban M Koput A Galar M New atherosclerosis risk factors in obese, hypertensive and diabetic children and adolescents Atherosclerosis 2003 167 275 286 10.1016/S0021-9150(03)00003-0 12818410

[B19] Lapolla A Piarulli F Sartore G Rossetti C Martano L Carraro P De Paoli M Fedele D Peripheral artery disease in type 2 diabetes: the role of fibrinolysis Thromb Haemost 2003 89 91 96 12540958

[B20] Rosito GA D'Agostino RB Massaro J Lipinska I Mittleman MA Sutherland P Wilson PW Levy D Muller JE Tofler GH Association between obesity and a prothrombotic state: the Framingham Offspring Study Thromb Haemost 2004 91 683 689 15045128 10.1160/th03-01-0014

[B21] Sobel BE Taatjes DJ Schneider DJ Intramural plasminogen activator inhibitor type-1 and coronary atherosclerosis Arterioscler Thromb Vasc Biol 2003 23 1979 1989 10.1161/01.ATV.0000091250.53231.4D 12920048

[B22] Bavenholm P Proudler A Silveira A Crook D Blomback M de Faire U Hamsten A Relationships of insulin and intact and split proinsulin to haemostatic function in young men with and without coronary artery disease Thromb Haemost 1995 73 568 575 7495060

[B23] Collet JP Montalescot G Vicaut E Ankri A Walylo F Lesty C Choussat R Beygui F Borentain M Vignolles N Thomas D Acute release of plasminogen activator inhibitor-1 in ST-segment elevation myocardial infarction predicts mortality Circulation 2003 108 391 394 10.1161/01.CIR.0000083471.33820.3C 12860898

[B24] Smith A Patterson C Yarnell J Rumley A Ben-Shlomo Y Lowe G Which hemostatic markers add to the predictive value of conventional risk factors for coronary heart disease and ischemic stroke? The Caerphilly Study Circulation 2005 112 3080 3087 10.1161/CIRCULATIONAHA.105.557132 16286603

[B25] Zietz B Drobnik W Herfarth H Buechler C Scholmerich J Schaffler A Plasminogen activator inhibitor-1 promoter activity in adipocytes is not influenced by the 4 G/5 G promoter polymorphism and is regulated by a USF-1/2 binding site immediately preceding the polymorphic region J Mol Endocrinol 2004 32 155 163 10.1677/jme.0.0320155 14765999

[B26] Anand SS Yi Q Gerstein H Lonn E Jacobs R Vuksan V Teo K Davis B Montague P Yusuf S Relationship of metabolic syndrome and fibrinolytic dysfunction to cardiovascular disease Circulation 2003 108 420 425 10.1161/01.CIR.0000080884.27358.49 12860914

[B27] Juhan-Vague I Pyke SD Alessi MC Jespersen J Haverkate F Thompson SG Fibrinolytic factors and the risk of myocardial infarction or sudden death in patients with angina pectoris. ECAT Study Group. European Concerted Action on Thrombosis and Disabilities Circulation 1996 94 2057 2063 8901651 10.1161/01.cir.94.9.2057

[B28] Juhan-Vague I Alessi MC Mavri A Morange PE Plasminogen activator inhibitor-1, inflammation, obesity, insulin resistance and vascular risk J Thromb Haemost 2003 1 1575 1579 10.1046/j.1538-7836.2003.00279.x 12871293

[B29] Loskutoff DJ Samad F The adipocyte and hemostatic balance in obesity: studies of PAI-1 Arterioscler Thromb Vasc Biol 1998 18 1 6 9445248 10.1161/01.atv.18.1.1

[B30] Ma LJ Mao SL Taylor KL Kanjanabuch T Guan Y Zhang Y Brown NJ Swift LL McGuinness OP Wasserman DH Prevention of obesity and insulin resistance in mice lacking plasminogen activator inhibitor 1 Diabetes 2004 53 336 346 10.2337/diabetes.53.2.336 14747283

[B31] Sahli D Eriksson JW Boman K Svensson MK Tissue plasminogen activator (tPA) activity is a novel and early marker of asymptomatic LEAD in type 2 diabetes Thromb Res 2009 123 701 706 10.1016/j.thromres.2008.07.015 18945481

[B32] Rai B Anand S Kharb SSm Atherosclerotic vascular disease and periodontal infection Pak J Medi Sci 2007 23 153

[B33] Alessi MC Poggi M Juhan-Vague I Plasminogen activator inhibitor-1, adipose tissue and insulin resistance Curr Opin Lipidol 2007 18 240 245 10.1097/MOL.0b013e32814e6d29 17495595

[B34] Alberti KG Zimmet P Shaw J The metabolic syndrome--a new worldwide definition Lancet 2005 366 1059 1062 10.1016/S0140-6736(05)67402-8 16182882

[B35] Auwerx J Bouillon R Collen D Geboers J Tissue-type plasminogen activator antigen and plasminogen activator inhibitor in diabetes mellitus Arteriosclerosis 1988 8 68 72 2449156 10.1161/01.atv.8.1.68

[B36] Jax TW Peters AJ Plehn G Schoebel FC Relevance of hemostatic risk factors on coronary morphology in patients with diabetes mellitus type 2 Cardiovasc Diabetol 2009 8 24 10.1186/1475-2840-8-24 19419582 PMC2688504

[B37] Juhan-Vague I Alessi MC Plasminogen activator inhibitor 1 and atherothrombosis Thromb Haemost 1993 70 138 143 8236090

[B38] Juhan-Vague I Roul C Alessi MC Ardissone JP Heim M Vague P Increased plasminogen activator inhibitor activity in non insulin dependent diabetic patients--relationship with plasma insulin Thromb Haemost 1989 61 370 373 2678583

[B39] Nordt TK Sawa H Fujii S Sobel BE Induction of plasminogen activator inhibitor type-1 (PAI-1) by proinsulin and insulin in vivo Circulation 1995 91 764 770 7828304 10.1161/01.cir.91.3.764

[B40] Galli-Tsinopoulou A Kyrgios I Maggana I Giannopoulou EZ Kotanidou EP Stylianou C Papadakis E Korantzis I Varlamis G Insulin resistance is associated with at least threefold increased risk for prothrombotic state in severely obese youngsters Eur J Pediatr 2010 21140274 10.1007/s00431-010-1370-9

[B41] Declerck PJ De Mol M Alessi MC Baudner S Paques EP Preissner KT Muller-Berghaus G Collen D Purification and characterization of a plasminogen activator inhibitor 1 binding protein from human plasma. Identification as a multimeric form of S protein (vitronectin) J Biol Chem 1988 263 15454 15461 2459123

[B42] Wiman B Almquist A Sigurdardottir O Lindahl T Plasminogen activator inhibitor 1 (PAI) is bound to vitronectin in plasma FEBS Lett 1988 242 125 128 10.1016/0014-5793(88)80999-2 2462509

[B43] Morioka S Makino H Shikata K Ota Z Changes in plasma concentrations of vitronectin in patients with diabetic nephropathy Acta Med Okayama 1994 48 137 142 7524268 10.18926/AMO/31126

[B44] Juhan-Vague I Morange PE Aubert H Henry M Aillaud MF Alessi MC Samnegard A Hawe E Yudkin J Margaglione M Plasma thrombin-activatable fibrinolysis inhibitor antigen concentration and genotype in relation to myocardial infarction in the north and south of Europe Arterioscler Thromb Vasc Biol 2002 22 867 873 10.1161/01.ATV.0000015445.22243.F4 12006404

[B45] Mathew M Tay E Cusi K Elevated plasma free fatty acids increase cardiovascular risk by inducing plasma biomarkers of endothelial activation, myeloperoxidase and PAI-1 in healthy subjects Cardiovasc Diabetol 2010 9 9 10.1186/1475-2840-9-9 20158910 PMC2837624

[B46] Goldberg RB Cytokine and cytokine-like inflammation markers, endothelial dysfunction, and imbalanced coagulation in development of diabetes and its complications J Clin Endocrinol Metab 2009 94 3171 3182 10.1210/jc.2008-2534 19509100

[B47] Viitanen L Pihlajamaki J Halonen P Lehtonen M Kareinen A Lehto S Laakso M Association of angiotensin converting enzyme and plasminogen activator inhibitor-1 promoter gene polymorphisms with features of the insulin resistance syndrome in patients with premature coronary heart disease Atherosclerosis 2001 157 57 64 10.1016/S0021-9150(00)00705-X 11427204

[B48] Eliasson MC Jansson JH Lindahl B Stegmayr B High levels of tissue plasminogen activator (tPA) antigen precede the development of type 2 diabetes in a longitudinal population study. The Northern Sweden MONICA study Cardiovasc Diabetol 2003 2 19 10.1186/1475-2840-2-19 14690546 PMC328088

[B49] Sobel BE Tilton L Neimane D Schnure J Increased tissue-type plasminogen activator: a facade in the fibrinolytic system in type 2 diabetes Coron Artery Dis 2005 16 31 35 10.1097/00019501-200502000-00006 15654197

[B50] Alexander CM Landsman PB Teutsch SM Haffner SM NCEP-defined metabolic syndrome, diabetes, and prevalence of coronary heart disease among NHANES III participants age 50 years and older Diabetes 2003 52 1210 1214 10.2337/diabetes.52.5.1210 12716754

[B51] Lempiainen P Mykkanen L Pyorala K Laakso M Kuusisto J Insulin resistance syndrome predicts coronary heart disease events in elderly nondiabetic men Circulation 1999 100 123 128 10402440 10.1161/01.cir.100.2.123

[B52] Anand SS Yusuf S Pogue J Ginsberg JS Hirsh J Relationship of activated partial thromboplastin time to coronary events and bleeding in patients with acute coronary syndromes who receive heparin Circulation 2003 107 2884 2888 10.1161/01.CIR.0000077530.53367.E9 12796128

[B53] Sakkinen PA Wahl P Cushman M Lewis MR Tracy RP Clustering of procoagulation, inflammation, and fibrinolysis variables with metabolic factors in insulin resistance syndrome Am J Epidemiol 2000 152 897 907 10.1093/aje/152.10.897 11092431

[B54] Arad Y Newstein D Cadet F Roth M Guerci AD Association of multiple risk factors and insulin resistance with increased prevalence of asymptomatic coronary artery disease by an electron-beam computed tomographic study Arterioscler Thromb Vasc Biol 2001 21 2051 2058 10.1161/hq1201.100257 11742884

[B55] Bonora E Kiechl S Willeit J Oberhollenzer F Egger G Bonadonna RC Muggeo M Carotid atherosclerosis and coronary heart disease in the metabolic syndrome: prospective data from the Bruneck study Diabetes Care 2003 26 1251 1257 10.2337/diacare.26.4.1251 12663606

[B56] Olijhoek JK vdGY Banga JD Algra A Rabelink TJ Visseren FL SMART Study Group The metabolic syndrome is associated with advanced vascular damage in patients with coronary heart disease, stroke, peripheral arterial disease or abdominal aortic aneurysm Eur Heart J 2004 25 342 348 10.1016/j.ehj.2003.12.007 14984924

[B57] Hsia J Bittner V Tripputi M Howard BV Metabolic syndrome and coronary angiographic disease progression: the Women's Angiographic Vitamin & Estrogen trial Am Heart J 2003 146 439 445 10.1016/S0002-8703(03)00227-8 12947360

[B58] Juhan-Vague I Morange PE Frere C Aillaud MF Alessi MC Hawe E Boquist S Tornvall P Yudkin JS Tremoli E The plasminogen activator inhibitor-1 -675 4G/5G genotype influences the risk of myocardial infarction associated with elevated plasma proinsulin and insulin concentrations in men from Europe: the HIFMECH study J Thromb Haemost 2003 1 2322 2329 10.1046/j.1538-7836.2003.00458.x 14629464

[B59] Meigs JB Mittleman MA Nathan DM Tofler GH Singer DE Murphy-Sheehy PM Lipinska I D'Agostino RB Wilson PW Hyperinsulinemia, hyperglycemia, and impaired hemostasis: the Framingham Offspring Study JAMA 2000 283 221 228 10.1001/jama.283.2.221 10634338

[B60] Suzuki J Ogawa M Muto S Itai A Hirata Y Isobe M Nagai R Effects of specific chemical suppressors of plasminogen activator inhibitor-1 in cardiovascular diseases Expert Opin Investig Drugs 2011 20 255 264 10.1517/13543784.2011.546784 21194395

[B61] Hamsten A Wiman B de Faire U Blomback M Increased plasma levels of a rapid inhibitor of tissue plasminogen activator in young survivors of myocardial infarction N Engl J Med 1985 313 1557 1563 10.1056/NEJM198512193132501 3934538

[B62] Thogersen AM Jansson JH Boman K Nilsson TK Weinehall L Huhtasaari F Hallmans G High plasminogen activator inhibitor and tissue plasminogen activator levels in plasma precede a first acute myocardial infarction in both men and women: evidence for the fibrinolytic system as an independent primary risk factor Circulation 1998 98 2241 2247 9826309 10.1161/01.cir.98.21.2241

[B63] Ladenvall P Johansson L Jansson JH Jern S Nilsson TK Tjarnlund A Jern C Boman K Tissue-type plasminogen activator -7,351C/T enhancer polymorphism is associated with a first myocardial infarction Thromb Haemost 2002 87 105 109 11848437

[B64] Jansson JH Olofsson BO Nilsson TK Predictive value of tissue plasminogen activator mass concentration on long-term mortality in patients with coronary artery disease. A 7-year follow-up Circulation 1993 88 2030 2034 8222095 10.1161/01.cir.88.5.2030

